# Brain Microvascular Endothelial Cell-Derived HMGB1 Facilitates Monocyte Adhesion and Transmigration to Promote JEV Neuroinvasion

**DOI:** 10.3389/fcimb.2021.701820

**Published:** 2021-08-31

**Authors:** Song-Song Zou, Qing-Cui Zou, Wen-Jing Xiong, Ning-Yi Cui, Ke Wang, Hao-Xuan Liu, Wen-Juan Lou, Doaa Higazy, Ya-Ge Zhang, Min Cui

**Affiliations:** ^1^ State Key Laboratory of Agricultural Microbiology, College of Veterinary Medicine, Huazhong Agricultural University, Wuhan, China; ^2^ Key Laboratory of Preventive Veterinary Medicine in Hubei Province, The Cooperative Innovation Center for Sustainable Pig Production, Wuhan, China; ^3^ Key Laboratory of Development of Veterinary Diagnostic Products, Ministry of Agriculture of the People’s Republic of China, Wuhan, China; ^4^ International Research Center for Animal Disease, Ministry of Science and Technology of the People’s Republic of China, Wuhan, China

**Keywords:** transmigration, adhesion, monocyte, HMGB1, Japanese encephalitis virus (JEV), neuroinvasion

## Abstract

Infection with Japanese encephalitis virus (JEV) induces high morbidity and mortality, including potentially permanent neurological sequelae. However, the mechanisms by which viruses cross the blood-brain barrier (BBB) and invade into the central nervous system (CNS) remain unclear. Here, we show that extracellular HMGB1 facilitates immune cell transmigration. Furthermore, the migration of immune cells into the CNS dramatically increases during JEV infection which may enhance viral clearance, but paradoxically expedite the onset of Japanese encephalitis (JE). In this study, brain microvascular endothelial cells (BMECs) were utilized for the detection of HMGB1 release, and leucocyte, adhesion, and the integrity of the BBB *in vitro*. Genetically modified JEV-expressing EGFP (EGFP-JEV) and the BBB model were established to trace JEV-infected immune cell transmigration, which mimics the process of viral neuroinfection. We find that JEV causes HMGB1 release from BMECs while increasing adhesion molecules. Recombinant HMGB1 enhances leukocyte-endothelium adhesion, facilitating JEV-infected monocyte transmigration across endothelia. Thus, JEV successfully utilizes infected monocytes to spread into the brain, expanding inside of the brain, and leading to the acceleration of JE onset, which was facilitated by HMGB1. HMGB1-promoted monocyte transmigration may represent the mechanism of JEV neuroinvasion, revealing potential therapeutic targets.

## Introduction

Japanese encephalitis virus (JEV) is a mosquito-borne, positive-sense single-stranded RNA virus (Misra and Kalita, 2010). JEV is an epidemic virus in the southern and eastern regions of Asia (Solomon et al., 2000; Misra and Kalita, 2010). The overall incidence of Japanese encephalitis (JE) is about 1.8 per 100,000. Approximately 20% of JE patients succumb to infection, and 50% of the survivors present with permanent neuropsychiatric sequelae (Solomon et al., 2000; Misra and Kalita, 2010). Numerous neurotropic pathogens affect blood-brain barrier (BBB) integrity, such as West Nile virus (WNV) and dengue virus (DENV) (Spindler and Hsu, 2012; Koyuncu et al., 2013). Pathologically, it has been suggested that impairment of the BBB is conclusively correlated with neuroinflammation. During JEV infection, dramatic BBB damage occurs, which is associated with Guillain-Barre syndrome (Wang et al., 2020). Currently, there are no effective therapeutics against JE (Misra and Kalita, 2010). Therefore, it is essential to investigate the pathways and mechanisms of JEV neuroinvasion.

As a dynamic interface of the central nervous system (CNS), the BBB is composed of closely packed fenestrated BMECs, supported by pericytes, astrocyte end-feet, neurons, and the extracellular matrix (Spindler and Hsu, 2012; Obermeier et al., 2013). The BBB manages transport and metabolism as a physical and physiological barrier, restricting the infiltration of immune cells into the brain (Obermeier et al., 2013; Li et al., 2015). The Transwell monolayer model mimics the BBB *in vitro*, and the integrity of the model can be reflected by the resistance measurement (TEER, transendothelial electrical resistance) *in vitro* (Eigenmann et al., 2013; Chen et al., 2014). During JEV infection, highly expressed proinflammatory cytokines and chemokines contribute to pathogenesis (Li et al., 2015). Increasing evidence suggests that recruitment and transmigration of leukocytes from the bloodstream to the CNS are involved in encephalitis (Man et al., 2007; Lim et al., 2011; Terry et al., 2012). Besides, cell adhesion molecules and their ligands promote immune cell trafficking, which has been confirmed by specific blockade assays or gene-deficient animal models (Miner and Diamond, 2016; Varatharaj and Galea, 2017).

There are several possible routes by which viruses invade the CNS: infection of BMECs, spread from the olfactory bulb, and immune cells acting as “Trojan horses” (Koyuncu et al., 2013; Suthar et al., 2013; Miner and Diamond, 2016). Infiltrated leukocytes present a paradoxical character in different diseases, especially monocytes, which can act as virus carriers disseminating the virus in the tissues or act as virus cleaner *via* activating immune responses. Nonetheless, the appearance of infected monocytes might act as an indicator of the severity of CNS disease (Charlier et al., 2009; Terry et al., 2012). Many viral infection models have shown that monocytes are well-suited viral vectors and exhibit the ability of transmigration as “Trojan horses” for viruses such as HIV-1, WNV, and ZIKV (Koyuncu et al., 2013; Ayala-Nunez et al., 2019; de Carvalho et al., 2019).

High-mobility group box 1 (HMGB1) is the most extensively studied HMG protein; normally located in the nucleus as a DNA chaperone (Romani et al., 1979; Muller et al., 2001; Stros, 2010). HMGB1 can actively translocate and be released from cells responding to stimuli (Kang et al., 2014; Hosakote et al., 2016). HMGB1 participates in cell recruitment, adhesion and migration as an adhesion molecule and a chemoattractant, and it is a damage-associated molecular pattern (DAMP) protein that initiates the immune response (Scaffidi et al., 2002; Rouhiainen et al., 2004; Kang et al., 2014). It has been confirmed that extracellular HMGB1 contributes to monocyte migration (Rouhiainen et al., 2004; Kang et al., 2014). Furthermore, extracellular HMGB1 initiates inflammatory pathways, leading to the production of multiple inflammatory factors (Scaffidi et al., 2002; Hosakote et al., 2016; Rayavara et al., 2018), which may damage the BBB.

In this study, JEV infection led to human brain microvascular endothelial cells (HBMECs) producing abundant HMGB1, which promoted JEV-infected monocytes transmigration, acting as “Trojan horses”, resulting in JEV neuroinvasion. These data provide insights into the correlation of leucocytes transmigration and JEV dissemination and may assist in JE treatment.

## Materials and Methods

### Mice and Virus

C57BL/6 mice were supplied by the Laboratory Animal Center of Huazhong Agricultural University, Wuhan, China. All work was performed following the Committee for Protection, Supervision, and the Control of Experiments on Animals guidelines of Huazhong Agricultural University. The JEV-P3 strain was employed in our previous research (Li et al., 2015) and 15 μl (5 × 10^4^ PFU) of viral inoculum was injected into the brains of 1-day suckling mice. After euthanization, the mouse (symptoms exhibition) brain was removed. Homogenized brains were suspended in Dulbecco’s modified Eagle’s medium (DMEM) at a concentration of 10% (wt/vol). After centrifugation, the debris was discarded and the supernatant was stored at −80°C until use. The baby hamster kidney fibroblast cell line (BHK-21) was used for viral titration by plaque assay.

### Viral Infection

Female C57BL/6 mice aged 6–8 weeks were classified into two groups: the control group (*n* = 6), which was injected with 50 μl of DMEM. In the JEV-infected group (*n* = 6), the mice were injected in the footpad with 10^5^ PFU or infected *via* intravenous injection (i.v.) with 5 × 10^6^ PFU of JEV. Mouse tissues were collected for tissue sectioning and RNA extraction.

Primary splenocytes and BMECs (HBMECs and bEnd.3 cells) were exposed to JEV at an MOI of 1 and incubated in DMEM at 37°C with 5% CO_2_ for 2 h. The cells were washed with PBS and then grown in culture medium. Virus-free cells were served as control.

### Cell Culture and Coculture

HBMECs were maintained in our laboratory and grown in DMEM containing 10% fetal bovine serum (FBS, Gibco, Grand Island, NY, USA) and endothelial cell growth supplement containing nonessential amino acids (Sigma, Ronkonkoma, NY, USA), minimum essential medium (Sigma, USA) vitamins, sodium pyruvate (Sigma, USA), 100 U/ml penicillin, and 100 mg/ml streptomycin sulfate at 37°C with 5% CO_2_. BHK-21 cells and bEnd.3 cells were maintained in our laboratory and grown in DMEM containing 10% FBS (Gibco, USA), 100 U/ml penicillin, and 100 mg/ml streptomycin sulfate at 37°C with 5% CO_2_. C6/36 cells were obtained from the Wuhan Institute of Virology, Chinese Academy of Sciences, cultured at 28°C with 5% CO_2_, using the same culture medium was used for the BHK-21 cells.

Primary splenocytes and peripheral blood mononuclear cells (PBMCs) were collected from healthy adult mice. After spinning down the red blood cells, cells were cultured at a density of 1 × 10^6^ cells/ml in DMEM containing 10% FBS, 100 U/ml penicillin, and 100 mg/ml streptomycin sulfate.

Primary splenocytes and PBMCs were treated with rHMGB1 (100 ng/ml, Sino Biological, Beijing, China) or infected with JEV (MOI = 1) and cultured at 37°C with 5% CO_2_. And RNA samples were collected at indicated times.

Primary splenocytes (treated or untreated) were incubated with the BMEC monolayer (JEV-infected or uninfected) in a 12-well plate for 2 h at 37°C with 5% CO_2_. Then the samples were collected for analysis at the indicated times. JEV-free or rHMGB1-free cells were served as control.

### Western Blotting

Cells were lysed in RIPA buffer containing protease inhibitor cocktail, homogenized, and centrifuged at 12,000×*g* and 4°C for 5 min. The protein concentration was determined by a BCA protein assay kit (Beyotime, Shanghai, China). Protein samples were separated by SDS-PAGE with 12% polyacrylamide gel. The proteins were transferred to polyvinylidene difluoride membranes (Bio-Rad, Richmond, CA, USA). Then, the proteins on the membranes were blocked for 2 h at room temperature in Tris-buffered saline with Tween 20 (TBST) containing 5% nonfat dry milk. The membranes were incubated overnight at 4°C with JEV-E protein monoclonal antibody (preserved in the laboratory) and the following antibodies: anti-ICAM-2, anti-beta-catenin, and anti-E-selectin (Proteintech, Wuhan, China); anti-HMGB1 (Novus Biologicals, Centennial, CO, USA); anti-VE-cadherin and anti-VCAM-1 (Abcam, Cambridge, MA, USA); and anti-Lamin A/C and anti-beta-actin (ABclonal, Wuhan, China). The membranes were washed with TBST and then incubated with horseradish peroxidase-conjugated (HRP) secondary antibodies. Enhanced chemiluminescence reagents (Bio-Rad, USA) were utilized to visualize the HRP-induced signal.

### Injection of Immune Cells Into the Brain

Purified CD3^+^ T cells, CD19^+^ B cells, and Ly6C^+^ monocytes were infected with JEV (MOI = 1) in DMEM for 2 h. The cells were then washed with PBS and incubated with JEV antiserum. Then, 1 × 10^5^ cells (virus-infected or uninfected) were injected into normal mouse brains. The JE onset time data were registered, and the JE mouse brains were removed for virus detection.

### Immunofluorescence

Ketamine-xylazine and PBS were used for the anesthetization and perfusion of symptomatic mice. The collected tissues were immediately fixed with 4% paraformaldehyde in an aseptic environment. All fixed tissues were embedded in paraffin for sectioning. In addition, the antigen was thermally retrieved in 0.01 M sodium citrate solution buffer.

Tissue sections were blocked in 5% BSA sealing fluid for 30 min at room temperature and incubated with anti-JEV-E protein monoclonal antibody overnight at 4°C. The sections were washed with PBS, incubated with the Alexa Fluor 488 labeled secondary antibody (Invitrogen, Grand Island, NY, USA) for 1 h, and nuclei were stained with 4′,6-diamidino-2-phenylindole (DAPI) for 3 min at room temperature. The tissue sections were sealed with glycerin. A fluorescence microscope was used to observe the sealed sections.

Highly express GFP-LFA-1 (ICAM-1 ligand) yeast cells were used to detect the ICAM-1 in the JEV-infected bEnd.3 cells (Zhang et al., 2018). The amount of the fluorescence (GFP) yeast cell represents the expression of ICAM-1 in the BMEC monolayer.

### BBB Monolayer Transwell Model

The BBB monolayer Transwell model (12-well, 3.0 μm pore size, Corning, NY, USA) was adopted in this study. Two hundred microliters of rat tail collagen (50 μg/ml, Sigma, USA) was used to enclose the upper Transwell chamber at room temperature for 1 h. After washed with PBS, 500 μl of DMEM (without phenol red, Sigma, USA) was added to the upper chamber for pre-equilibration at 37°C for 1 h. BMECs (5 × 10^5^ bEnd.3 cells) were cultured in the upper chamber with a total volume of 500 μl of culture medium (without phenol red, Sigma, USA) at 37°C and 5% CO_2_ for approximately 24 h. Monolayer leakage was monitored for 4 h, and FITC-dextran (10 kD and 70 kD, Sigma-Aldrich, USA) was added for the permeability measurement of the BBB monolayer model.

### Electric Cell-Substrate Impedance Sensing (ECIS)

Electrode plates were equilibrated in 500 µl DMEM overnight at 37°C. Then, 350 µl of bEnd.3 cells was added to each well at a density of 2 × 10^6^/ml. Until the impedance stabilization, real-time impedance changes were measured (Applied BioPhysics, Troy, NY, USA).

### Cell Transmigration

Virus was added to the upper Transwell chamber to infect the bEnd.3 cells (MOI = 1) for 2 h in DMEM (without phenol red, Sigma, USA). In addition, primary splenocytes were infected with virus or treated with rHMGB1 (100 ng/ml). After washing by PBS, the Transwell chamber was refreshed with new culture medium. The treated splenocytes (5 × 10^5^ cells) were added to the upper chamber and cocultured with the monolayer of infected bEnd.3 cells for 24 h at 37°C and 5% CO_2_. Then, the transmigrated cells were collected in the lower chamber and analyzed by flow cytometry.

Transendothelial electrical resistance (TEER; ohm.cm^2^) (Millicell, ERS-2,Millipore, Billerica, MA), a criterion used to evaluate the permeability of monolayer models *in vitro*, was monitored and recorded at the indicated times (0 h, 6 h, 12 h, 18 h, and 24 h).

### Flow Cytometry and Quantitative Real-Time PCR Analysis

Splenocytes were stained with the combination of mAbs conjugated with FITC, PE, PE-Cy7, APC-Cy7, PB, and APC. For cell surface marker staining, splenocyte suspensions were incubated with the appropriate Abs, anti-CD3, anti-CD11b, anti-Ly6C, and anti-CD19 in PBS buffer (pH = 7.4) containing 0.2% BSA (BioSharp, China), at 4°C for 30 min. PBS was provided for double washing (400×*g*, 5 min, 4°C), and cell suspension. Cell identification and separation were achieved by flow cytometry with FACS Calibur (BD Biosciences, Billerica, MA, USA) system or Beckman CytoFlex (Beckman Coulter, Carlsbad, CA, USA), and CytExport 2.0 CellQuest Pro software were used for data analysis. The EGFP-JEV is utilized is to intracellular JEV detection.

Total RNA was extracted with TRIzol reagent (Invitrogen, USA). One microgram of RNA was used to synthesize cDNA with a ReverTra Ace RT-PCR RT kit (Toyobo, Osaka, Japan) following the manufacturer’s instructions. SYBR Green (Invitrogen, USA) was employed for quantitative real-time PCR using StepOne Plus and StepOne Software v2.2.2 (Applied Biosystems, Foster City, CA, USA). The relative expression of the JEV-C gene was normalized to the level of the beta-actin. The pcDNA3.0-HA/JEV-C gene plasmid served as a template for generating a standard curve to quantify JEV copy numbers. The real-time PCR primers were listed in **Supplementary Table S1**.

### Statistical Analysis

All experiments were repeated at least three times. The data are expressed as the means ± SEM. The data were analyzed by Student’s *t*-test or one-way analysis of variance followed by Tukey’s *post-hoc* tests. Graphs were plotted and analyzed using GraphPad Prism software (v7.0; GraphPad, La Jolla, CA, USA).

## Results

### JEV Infection Caused HMGB1 Cytoplasmic Translocation and Secretion From HBMEC

**Figure 1:**
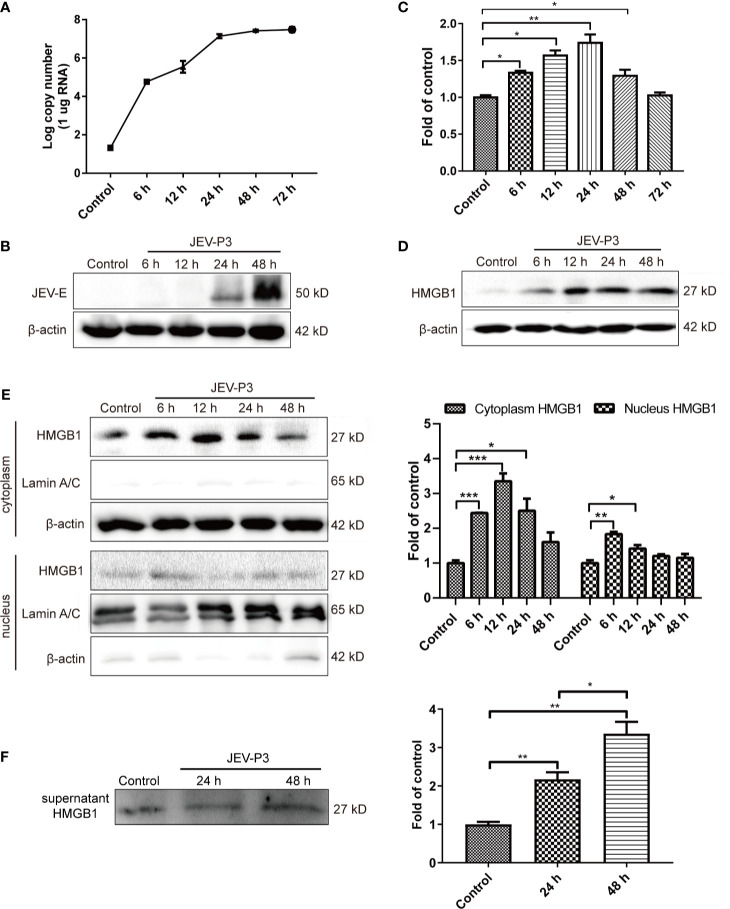
JEV-induced fluctuation of HMGB1 in HBMECs. HBMECs were infected with JEV-P3 at an MOI of 1, and total cell protein and RNA samples were collected at the indicated times to measure JEV replication in HBMECs by real-time PCR **(A)** and Western blotting using an anti-JEV-E protein monoclonal antibody **(B)**. HMGB1 expression was measured by real-time PCR **(C)** and Western blot **(D)** at the indicated times during JEV infection. JEV free cells were served as control. **(E)** Total cytoplasmic and nuclear proteins were extracted from JEV-infected HBMECs at 0 h (Control), 6 h, 12 h, 24 h, and 48 h postinfection. HMGB1 protein expression was measured by Western blotting, with beta-actin as the internal control for protein integrity and Lamin A/C was assessed in the nuclear extract, and quantitatively analyzed as the fold change relative to the control. **(F)** HBMEC culture supernatant was collected at indicated times after virus infection (24 h, and 48 h). Supernatant HMGB1 was measured by Western blotting and quantitatively analyzed as the fold change relative to the control (JEV-free cell culture supernatant). The experiments are repeated at least three times. The data are expressed as the means ± SEM. *p < 0.05, **p < 0.01, and ***p < 0.001.

HBMECs were infected with JEV at an MOI of 1, and the expression of JEV-E protein was measured by Western blotting. JEV replicated in HBMECs (**Figure 1A**), and abundant intracellular JEV-E protein was observed at 24 and 48 h (**Figure 1B**). JEV infection caused a dramatic increase in HMGB1 expression at both the mRNA and protein levels (**Figures 1C, D**). In addition, HMGB1 was upregulated in mouse BMECs (bEnd.3 cell line) during JEV infection (**Figure S1A**). These results demonstrate that JEV infection induced upregulation of HMGB1 expression in BMECs.

The biological functions of HMGB1 are dominated by its expression and subcellular localization (Deng et al., 2019). Thus, the cellular distribution and release of HMGB1 were determined. HMGB1 was mainly located in the nucleus of uninfected HBMECs and expressed at a low level, but its expression was significantly increased in the cytoplasm of JEV-infected HBMECs at 24 h (**Figure S1B**), suggesting the translocation of HMGB1 from the nucleus to the cytoplasm. To confirm this translocation, the protein was extracted separately from the nucleus and cytoplasm, and the HMGB1 level was detected (**Figure 1E**). The results showed a significant increase of HMGB1 in the cytoplasm after JEV infection, reaching a peak at 12 h and then gradually declining from 24 h to 48 h. The expression of HMGB1 in the nucleus was increased at 6 h. Accumulation of HMGB1 in the cytoplasm may actively initiate HMGB1 secretion. The detection of secreted HMGB1 revealed approximately 2- and 3.5-fold increase in HMGB1 released from JEV-infected cells at 24 and 48 h, respectively (**Figure 1F**).

Taken together, these data suggested that JEV induces the upregulation and translocation of HMGB1, which is subsequently released from cells.

### JEV Infection Induced the Activation of BMECs and an Increase in the Expression of Adhesion Molecules

**Figure 2:**
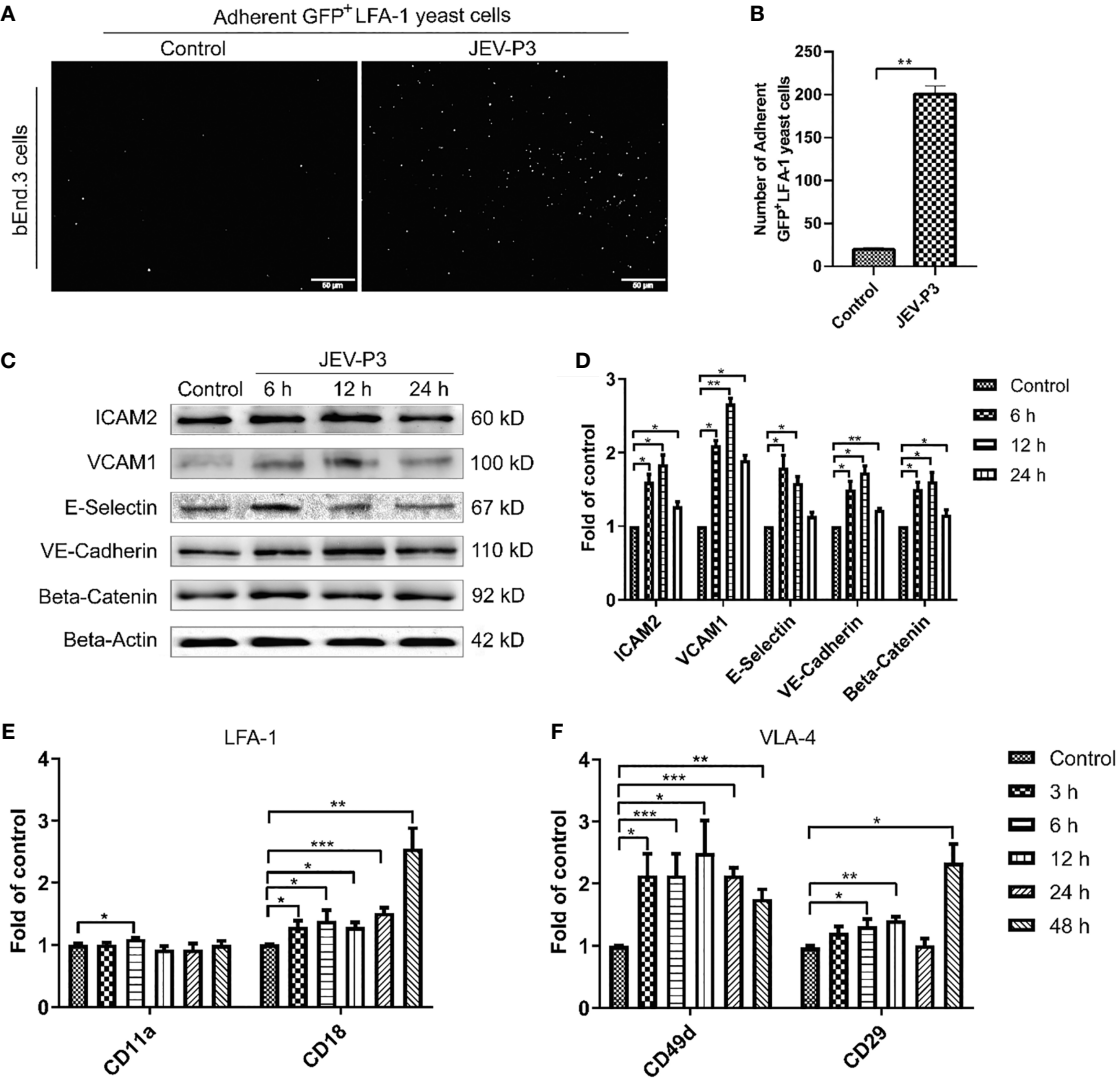
JEV infection upregulated adhesion molecules expression in bEnd.3 cells, and rHMGB1 increased the expression of integrin ligands in splenocytes. **(A)** ICAM-1 expression level was detected in the JEV-infected bEnd.3 cells at 6 h. Representative images showing the binding of highly expressed GFP^+^ LFA-1 yeast cells. **(B)** Statistical analysis of fluorescence was performed, and the result represents the expression levels of ICAM-1 in bEnd.3 cells. **(C)** Detection of ICAM-2, VCAM-1, E-selectin (CD62E), VE-cadherin, and beta-catenin expression levels in JEV-infected bEnd.3 cells were determined by Western blotting. Protein samples were collected at 0 h (Control), 6 h, 12 h, and 24 h. **(D)** The protein expressions reported in panel **(C)** were normalized to that of beta-actin and quantitatively analyzed as the fold change relative to the control. The expression levels of LFA-1 (CD11a and CD18) **(E)** and VLA-4 (CD49d and CD29) **(F)** in rHMGB1-treated (100 ng/ml) mouse splenocytes, determined by real-time PCR at 0 h (Control), 3 h, 6 h, 12 h, 24 h, 48 h. The scale bar for **(A)** is 50 μm. The experiments were repeated at least three times. The data are expressed as the means ± SEM. *p < 0.05, **p < 0.01, and ***p < 0.001.

BMECs are critical to the formation of the BBB and the maintenance of its barrier function. High expression of adhesion molecules and integrin ligands is necessary for circulating cell adhesion to the BBB endothelium, which may facilitate cell infiltration into the CNS. In this study, yeast cells that highly express GFP-LFA-1 (ICAM-1 ligand) were used to detect ICAM-1 expression and LFA-1-ICAM-1-mediated interactions between yeast cells and JEV-activated endothelial cells (Zhang et al., 2018). The JEV-infected bEnd.3 monolayer accommodated more GFP^+^ LFA-1 yeast cells than the control monolayer (**Figures 2A, B**), which suggested that JEV induced upregulation of ICAM-1 in the bEnd.3 cells. Western blotting confirmed the increase in adhesion molecules on endothelial cells following infection, including VCAM-1, ICAM-2, E-selectin, VE-cadherin, and beta-catenin (**Figures 2C, D**). Since HMGB1 is released from BMECs, which may affect circulating immune cells, rHMGB1 was used to treat the isolated splenocytes *in vitro*, and the expression of adhesion molecules was measured. As expected, treatment with rHMGB1 (100 ng/ml) upregulated LFA-1 and VLA-4 on mouse splenocytes (**Figures 2E, F**), which act as receptors of ICAM-1 and VCAM-1.

In addition, the expression of ICAM-1 and VCAM-1 in HBMECs was increased after JEV infection (**Figures S2A, B**). With the treatment of rHMGB1, an upregulation was also observed in the expression of LFA-1 (CD11a and CD18) and VLA-4 (CD49d and CD29) in human THP-1 cells (**Figures S2C, D**). Furthermore, upregulation of ICAM-1 and VCAM-1 was found in JEV-infected mouse brains (**Figures S3A, B**) and was coupled with an increase of LFA-1 and VLA-4 expression in the PBMCs (**Figures S3C, D**).

All these results suggested that JEV infection upregulated adhesion molecules on BMECs and that HMGB1 also induced an increase in integrin ligands on circulating immune cells, which may contribute to immune cells binding to the BBB endothelium.

### Extracellular HMGB1 Promoted the Adhesion of Immune Cells to the Endothelium

**Figure 3:**
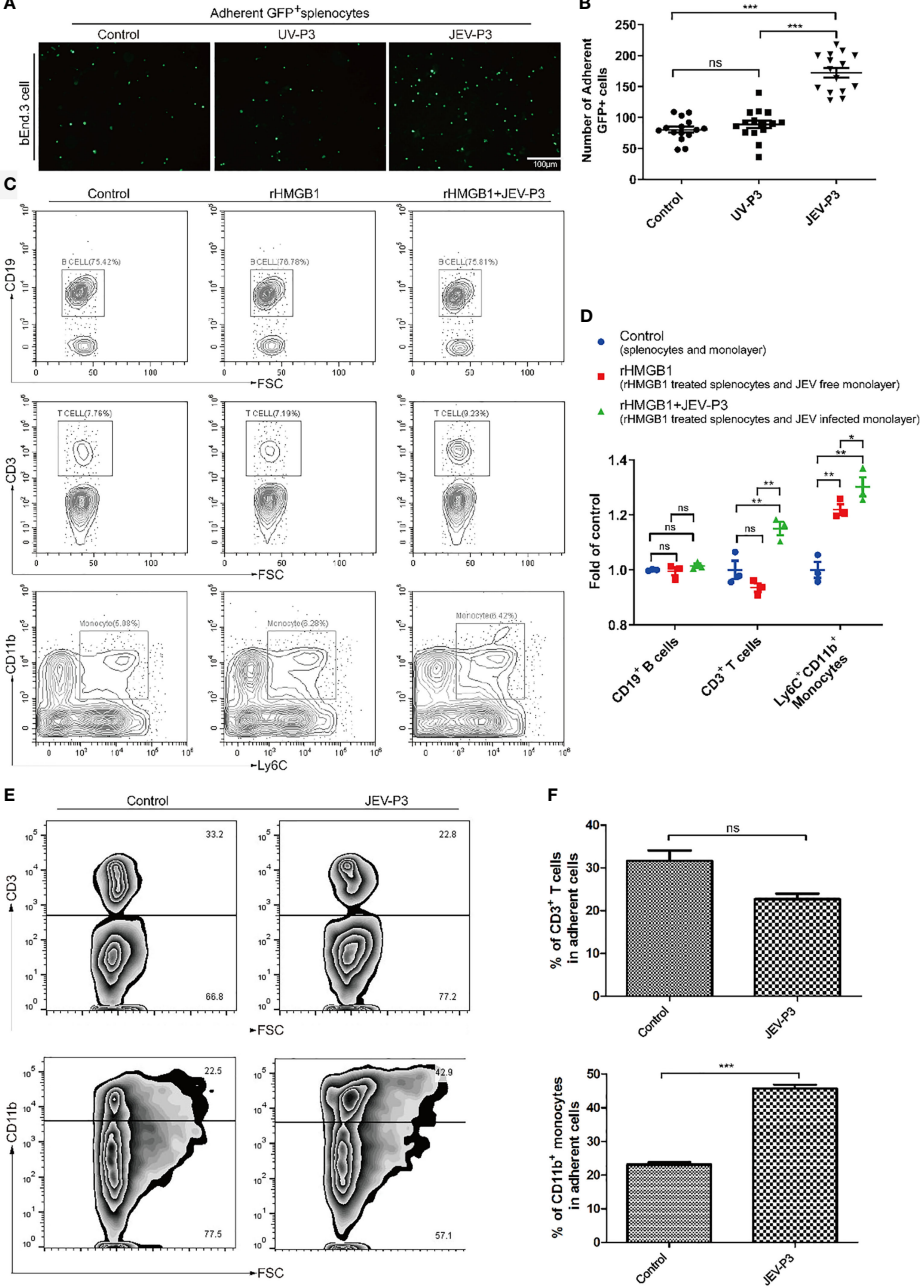
HMGB1 promoted immune cell adhesion to the BMEC monolayer. **(A)** GFP^+^ splenocytes were incubated with JEV-P3/UV-P3-infected bEnd.3 cell monolayers for 2 h; the GFP^+^ splenocytes were obtained from transgenic mice. After washing with PBS, the cells were fixed with 4% paraformaldehyde and then observed by fluorescence microscopy. The fluorescence represents the number of adherent splenocytes. JEV free monolayers were served as control. **(B)** Statistical analysis of the GFP^+^ splenocytes binding to the bEnd.3 cell monolayer of **(A)**. **(C)** Mouse splenocytes were treated with rHMGB1 (100 ng/ml) for 2 h. Then, splenocytes (rHMGB1 treated or untreated) were incubated with the bEnd.3 cell monolayers (JEV infected or uninfected) for 2 h. After gentle washing with PBS, the adherent splenocytes were collected, and the amount of CD19^+^ B cells, CD3^+^ T cells, and Ly6C^+^CD11b^+^ monocytes was analyzed by flow cytometry. Untreated splenocytes and uninfected monolayers were served as control. **(D)** Statistical analysis of the binding splenocytes to the bEnd.3 cell monolayer as reported in **(C)**. **(E)** Purified CD3^+^ T cells and CD11b monocytes (obtained from JEV-infected mice, tail vein injection **(F)**, 3 dpi) were inoculated onto the virus-infected bEnd.3 cell monolayer and incubated for 2 h. After washing with PBS, the bound cells were detected by flow cytometry. The right panels show the results of the statistical analysis of CD3^+^ T cells and CD11b monocytes binding to the bEnd.3 cell monolayer of **(E)**. JEV free bEnd.3 cell monolayers were served as control. The scale bar for **(A)** is 100 μm. These experiments were repeated at least three times. The data are expressed as the means ± SEM. *p* > 0.05 (ns, no significant difference), *p < 0.05, **p < 0.01, and ***p < 0.001.

Leukocyte-endothelium adhesion is indispensable for the infiltration of cells into the CNS. bEnd.3 monolayers were primed with live JEV-P3 or UV-deactivated JEV-P3. More GFP^+^ splenocytes, isolated from GFP-transgenic mice, were bound to the JEV-infected monolayer group compared with the control (uninfected monolayer) and UV-deactivated virus groups (**Figures 3A, B**). Moreover, HMGB1 was overexpressed in 293T cells (data not shown). The supernatant from these HMGB1-overexpressing 293T cells was collected to treat THP-1 cells. More supernatant HMGB1-treated THP-1 cells were adherent to the virus-infected HBMECs compared with the untreated THP-1 cells (**Figure S4A**). To investigate whether HMGB1 stimulate immune cell adherence to the BBB endothelium, mouse splenocytes were treated with only rHMGB1 and then added to a bEnd.3 monolayer. After 2 h of incubation, the cells were gently washed and subjected to flow cytometry analysis. The results showed that rHMGB1 treatment led to more Ly6C^+^CD11b^+^ monocytes binding to the monolayer, but there were no significant effects on CD3^+^ T cells or CD19^+^ B cells (**Figures 3C, D**). Additionally, rHMGB1 treatment stimulated more CD3^+^ T cells and Ly6C^+^CD11b^+^ monocytes adhering to the JEV-primed endothelial monolayer (**Figures 3C, D**). Furthermore, CD3^+^ T cells and CD11b^+^ monocytes were sorted from the JEV-infected mice at 3 dpi and then added to a BMEC monolayer. JEV infection dramatically enhanced the adherence of CD11b^+^ monocytes but not CD3^+^ T cells to the BMECs (**Figures 3E, F**). These results indicated that the upregulation of adhesion molecules triggered by JEV and the extracellular HMGB1 can promote monocyte binding to the BBB, which may potentiate monocyte crossing the BBB.

### Extracellular HMGB1 Facilitated Transendothelial Migration of JEV-Infected Monocytes

**Figure 4:**
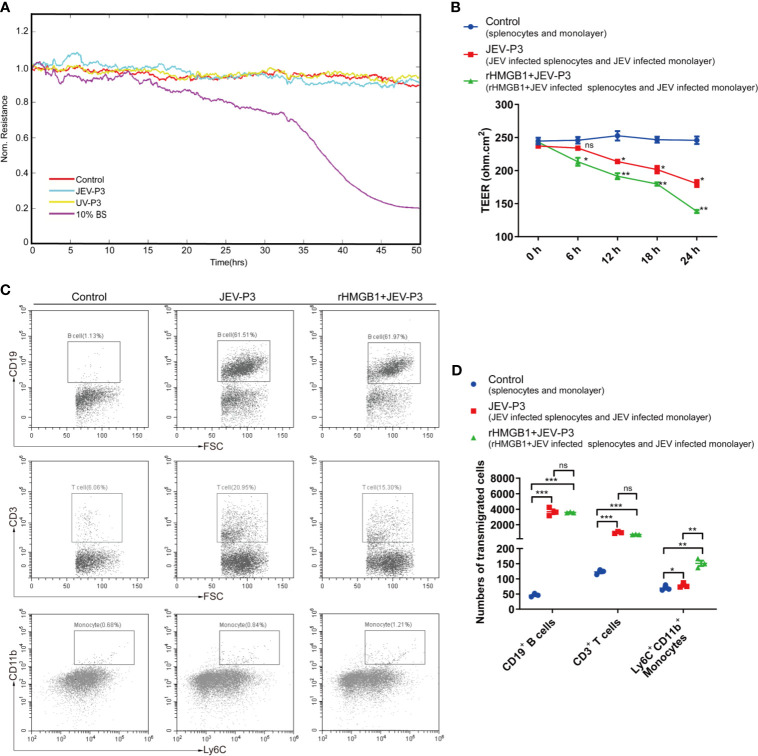
HMGB1 facilitated immune cell transmigration during infection. **(A)** Real-time measurement of the JEV-P3/UV-P3 effect on the tight junction between bEnd.3 cells *in vitro*. The electrical resistance value represents the tight junction integrity of the bEnd.3 cells. TEER was measured at 4 kHz. bEnd.3 cells were exposed to JEV-P3, UV-P3, and 10% BS (the supernatant of JEV-infected mouse brain). Each line represents experimental repeats as measured in three wells of cells. Increased resistance is positively correlated with the barrier function of bEnd.3 cells. **(B)** bEnd.3 cells were cultured in the collagen-covered upper Transwell chamber in culture medium (without phenol red) for 24 h. Then, the permeability and TEER of the BMEC monolayer models were measured. Mouse splenocytes (5 × 10^5^) were added to the upper chamber and cocultured with the monolayer for 24 h. Then, the transmigrated cells in the lower chamber were collected and counted by flow cytometry. The TEER value of control (splenocytes cocultured with monolayer), JEV-P3 (JEV infected splenocytes cocultured with JEV infected monolayer) and rHMGB1+JEV-P3 (rHMGB1 was added in the cocultured system of JEV infected splenocytes and JEV infected monolayer) models were measured at the indicated times (0 h, 6 h, 12 h, 18 h, and 24 h). **(C)** After splenocytes were cocultured with the bEnd.3 cell monolayer for 24 h, the transmigrated cells (obtained from the lower chamber), including CD19^+^ B cells, CD3^+^ T cells, and Ly6C^+^CD11b^+^ monocytes, were collected and analyzed by flow cytometry. rHMGB1 free and JEV free groups were served as control. **(D)** Statistics of transmigration cells in the lower chamber of panel **(C)**. The experiments were repeated at least three times. The data are expressed as the means ± SEM. *p* > 0.05 (ns, no significant difference), *p < 0.05, **p < 0.01, and ***p < 0.001.

Notably, JEV infection did not have a significant effect on the integrity of the endothelium *in vitro*, and there was no difference between the virus and the UV-inactivated virus treatments (**Figure 4A**). However, treatment with JEV-infected mouse brain supernatant (10%) led to a loss of integrity in the BMEC monolayer (**Figure 4A**), which suggested that JEV does not disrupt the BBB, but the subsequent inflammatory reactions may result in BBB damage.

To further elucidate the role of extracellular HMGB1 in leucocyte migration during JEV infection, a Transwell insert was covered with bEnd.3 cells (**Figure S5A**). The cells formed a tight monolayer, as expected, reaching the standard level of confluence. Few FITC-dextran (10 kD and 70 kD) was detected in the lower chamber compared with the upper chamber, indicating robust membrane impermeability (**Figure S5B**). The integrity of the BBB, evaluated by TEER, remained stable over 24 h (>200 ohm.cm^2^) in the control monolayers (**Figure 4B**). Quantification of the TEER showed that the exposure to rHMGB1 exacerbated the destruction of the monolayer during JEV infection compared with the effect on the virus-infected cells (**Figure 4B**). To confirm that HMGB1 promotes leucocyte migration, the Transwell model was used for a transmigration assay. Flow cytometry analysis indicated that JEV infection triggered the transmigration of Ly6C^+^CD11b^+^ monocytes, CD3^+^ T cells, and CD19^+^ B cells (**Figures 4C, D**), accompanied by a decrease in the TEER (**Figure 4B**). Moreover, rHMGB1 led to significantly more Ly6C^+^CD11b^+^ monocyte transmigration upon JEV infection but had no effects on CD3^+^ T cells and CD19^+^ B cells compared with the effect on the virus-infected cells (**Figures 4C, D**). These results indicated that HMGB1 can exacerbate BBB fluctuation and monocyte transmigration during JEV infection.

**Figure 5:**
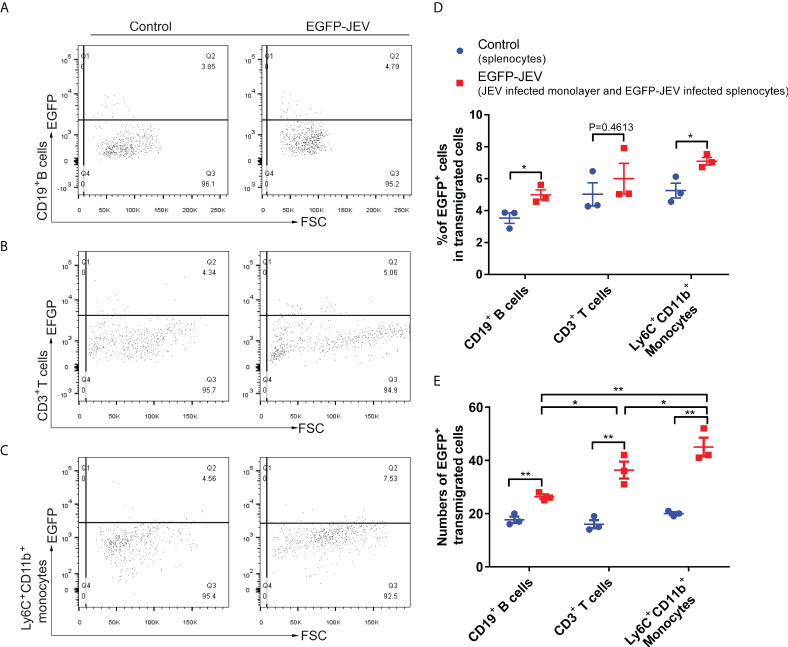
Virus-carrying splenocyte transmigration *in vitro*. **(A–C)** JEV-infected bEnd.3 cell monolayers were cocultured with EGFP-JEV-infected splenocytes (5 × 10^5^) for 24 h, and the transmigrated cells (lower chamber) were collected and measured by flow cytometry. An enhanced sensitivity measure at 488 nm was performed for the detection of intracellular EGFP-JEV in CD19^+^ B cells, CD3^+^ T cells, and Ly6C^+^CD11b^+^ monocytes by flow cytometry. EGFP-JEV free cells were the nonspecific control. **(D**, **E)** The statistical analysis of EGFP-positive cells in the transmigrated cells in the lower chamber reported in **(A**–**C)**. The experiments were repeated at least three times. The data are expressed as the means ± SEM. *p* > 0.05 (ns, no significant difference), *p < 0.05 and **p < 0.01.

To discover which cells act as virus carriers, JEV with an EGFP tag (EGFP-JEV) was applied to visualize cell transmigration. There was an increased percentage of EGFP-positive Ly6C+CD11b+ monocytes, CD3+ T cells, and CD19+ B cells that transmigrated, compared with the control cells (**Figures 5A–C**). Furthermore, there were significantly more transmigrated JEV-positive (EGFP+Ly6C+CD11b+) monocytes than transmigrated JEV-positive T cells (EGFP+CD3+) or B cells (EGFP+CD19+) (**Figures 5D, E**).

These data suggested that extracellular HMGB1 promotes cell transmigration, especially monocytes, which serve as “Trojan horses” during JEV neuroinvasion.

### JEV-Infected Immune Cells Disseminate JEV to the was Brain Correlated With JE in Mouse

**Figure 6:**
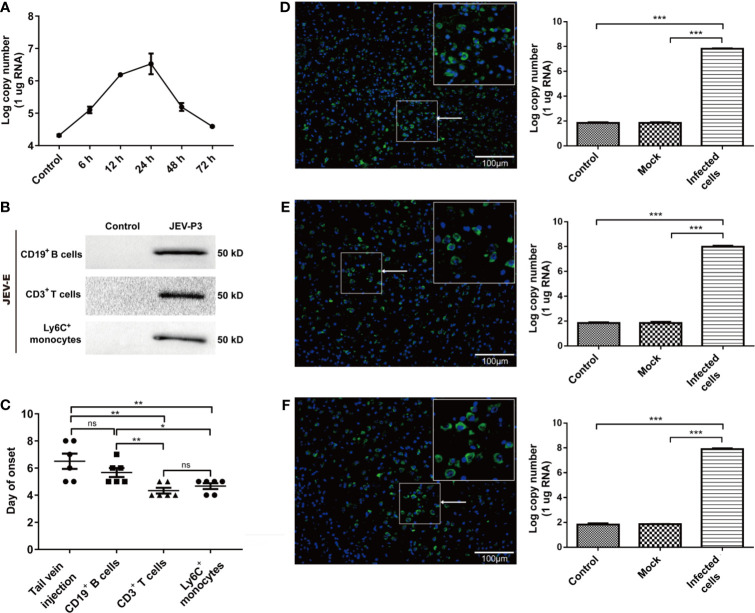
JEV-infected immune cells facilitating virus dissemination in the CNS were affiliated with JE in mice. **(A)** The replication of JEV in mouse splenocytes from 0 h (Control) to 72 h was measured by real-time PCR. **(B)** Purified CD3^+^ T cells, CD19^+^ B cells, and Ly6^+^ monocytes were exposed to JEV-P3, which was detected by Western blotting using an anti-JEV-E protein antibody. JEV free cells were served as control. **(C)** The statistical analysis of JE onset after JEV-infected CD19^+^ B cells, CD3^+^ T cells, and Ly6C^+^ monocytes were injected into healthy adult mice brains, compared with the JE onset in the tail vein injection group. After the intracranial injection of PBS (Control), JEV-infected (Infected cells) or uninfected (Mock) Ly6C^+^ monocytes **(D)**, CD3^+^ T cells **(E)**, and CD19^+^ B cells. **(F)** Immunofluorescence images of the JE mice brains, which showed cells stained for DNA (blue, DAPI) and JEV (green, JEV-E protein). JEV replication in mouse brains was measured by real-time PCR. The scale bar for **(D**–**F)** is 100 μm. The experiments were repeated at least three times. The data are expressed as the means ± SEM. *p* > 0.05 (ns, no significant difference), *p < 0.05, **p < 0.01, and ***p < 0.001.

Mouse splenocytes were isolated and subjected to JEV infection. Real-time PCR showed that JEV replication reached a peak at 24 h (**Figure 6A**). The purified Ly6C^+^ monocytes, CD3^+^ T cells, and CD19^+^ B cells were exposed to JEV and assessed by Western blot. The findings showed the ability of the virus to replicate in the purified cells (**Figure 6B**). The results in **Figure 5** indicated monocyte could carry JEV across the BBB *in vitro*. To study virus-infected leucocyte and virus CNS dissemination, virus-infected cells were incubated with the anti-JEV serum to neutralize nonspecifically adhering viruses. Thus, 1 × 10^5^ cells were intracranially injected into the brain to simulate cell-associated JEV dissemination *in vivo*. As expected, the injection of virus-infected cells caused mouse disease symptoms (emaciation, seizures, motion disorders, and paralysis), same as that in the group injected with the virus *via* the tail vein. Furthermore, the initial appearance of JE was earlier in the mice with the intracranial injection of JEV-infected Ly6C^+^ monocytes and CD3^+^ T cells than in the mice injected with JEV-infected CD19^+^ B cells (**Figure 6C**). All types of virus-infected cells induced JE in mice and caused greater virus replication in the brain compared with the control (PBS injection) and mock groups (virus-free cell injection) (**Figures 6D**–**F**). Moreover, the onset time of JE was positively associated with the quantity of JEV-infected immune cells injected (data not shown). These data indicated that cell-associated JEV promotes viral dissemination, and eventually leads to neurological disease.

The natural route of infection was also mimicked by JEV injection in the footpad of C57BL/6 mice. Tissue samples were collected from mouse cerebrum, olfactory bulb, and spinal cord after infection, and the viral loads were determined by real-time PCR and immunofluorescence. The results showed that there was a higher number of the JEV particles in the cerebrum than in the olfactory bulb or spinal cord (**Figures S6A–C**). These results suggested that CNS dissemination of JEV presumably occurs through the blood.

Together, these data indicated that JEV-infected immune cells, especially monocytes, serves as “Trojan horses” carrying JEV to the brain and contribute to JE onset in mouse.

## Discussion

HMGB1 is a DNA-binding, intracellular transcription-regulating protein (Kang et al., 2014). Cell activation or necrosis induces HMGB1 translocation to the cytoplasm and its release into the extracellular space (Hosakote et al., 2016), which has been described as a DAMP factor that initiates inflammatory responses and regarded as a cell migration mediator (Lotze and Tracey, 2005; Kang et al., 2014). In this study, we demonstrated that JEV infection triggered HMGB1 release from BMECs. BMEC-derived HMGB1 promoted immune cells binding to the BBB endothelium and transmigrating into the CNS as “Trojan horses”. Therefore, HMGB1, as a mediator of intercellular adhesion and transmigration, promotes virus neuroinvasion and contributes to the pathogenesis of JEV, which was associated with JEV-infected monocytes.

Previous studies have reported that viral infection induces the translocation and secretion of HMGB1 (Wang et al., 2006; Hosakote et al., 2016). Additionally, HMGB1 is important to cell migration, particularly monocyte transmigration (Rouhiainen et al., 2004; Kang et al., 2014). This investigation primarily illustrated that JEV infection induced the release of HMGB1 from BMECs. JEV infection also induced the initiation of cell activation and upregulated the expression of adhesion molecules, such as ICAM-1 and VCAM-1. In addition, HMGB1 upregulated the expression of LFA-1 and VLA-4 in immune cells. Increase in adhesion molecules and HMGB1 level facilitates leukocyte binding to virus-infected monolayers and can promote immune cell CNS transmigration (Ley and Reutershan, 2006; Lai et al., 2012; Ayala-Nunez et al., 2019). However, the interaction between virus-infected leukocytes and BBB homeostasis remains unclear. Our results from a previous study revealed that JEV itself is not the leading cause of the tight junction loss between endothelial cells during early infection. In contrast, supernatant of JE brain, containing proinflammatory factors, dramatically destroyed the integrity of the BBB monolayer *in vitro*, supporting the idea that a systemic inflammatory response disrupts the BBB (Li et al., 2015; Mustafa et al., 2019).

It has been shown that during HIV/WNV infection, as the virus carriers, immune cells (T cells, monocytes) are recruited to the BBB surface for CNS infiltration (Terry et al., 2012; Koyuncu et al., 2013). Similarly, monocytes also act as JEV carriers and transmigrate to the CNS, causing neuroinfection. It has been suggested that extracellular HMGB1 may activate immune cells to produce inflammatory cytokines (Rayavara et al., 2018). Moreover, previous evidence has shown that HMGB1 is directly associated with the breakdown of the BBB *in vitro* (Festoff et al., 2016). Monocyte migration is probably different from that of T cells in response to HMGB1, which showed a distinct difference in transmigration efficiency in our model. Furthermore, our data suggested that HMGB1 accelerates the breakdown of the BBB and immune cell infiltration during JEV infection, which was reflected by a decrease in the TEERs and an increase in the amount of migrating cells. All the results from our study suggest that HMGB1 facilitates the endothelial adhesion and transmigration of monocytes during JEV infection, including specific and nonspecific binding and migration cells. However, neither the BBB monolayer model nor the *in vitro* Transwell model could fully represent the intact BBB *in vivo* (Helms et al., 2016). More comprehensive *in vivo* or *in vitro* BBB models are being developed or sought for the study of JEV-infected monocytes with HMGB1-mediated effects in the early stage of viral neuroinvasion.

Neurotropic viruses may spread through multiple pathways to achieve CNS invasion, including endothelial cell infection and the “Trojan horses”. The intracranial injection of virus-infected cells is performed to link that cell-associated JEV could promote viral dissemination in the brain. However, direct evidence of cell migration *in vivo* is still lacking, and further *in vivo* studies using JEV infection models to explore the mechanism of natural infection are necessary. It has been reported that virus-infected monocytes acting as Trojan horse-like carriers contribute to virus dissemination in the CNS, enhancing viral persistence (O'Connor et al., 2018; Ayala-Nunez et al., 2019). Notably, it has been suggested that the migration of T cells into the CNS may be regulated by the transmigration of monocytes (Savarin et al., 2010; Man et al., 2012; Netland and Bevan, 2013). Therefore, monocytes may act as an important mediator in viral spread towards neural tissues, and further study is needed to evaluate the relative contribution of monocytes to JEV neuroinvasion.

**Figure 7:**
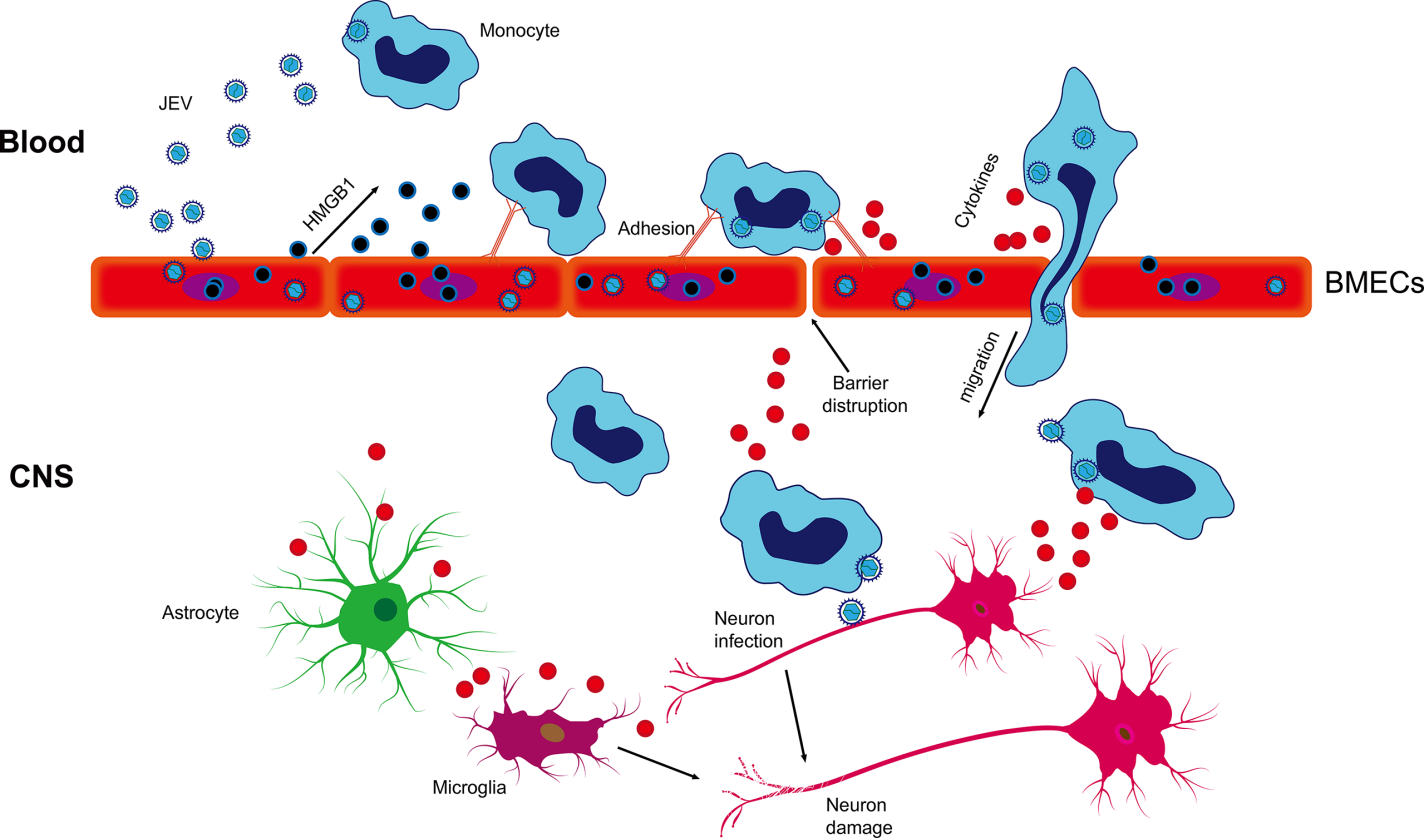
Schematic description of BMEC-derived HMGB1 contributing to JEV-infected monocyte transendothelial migration. JEV induced HMGB1 release from BMECs and upregulated the expression of adhesion molecules, which showed enhanced leukocyte-endothelium adhesion accompanied by promoted JEV-infected monocyte transendothelial migration and BBB fluctuation. JEV-infected monocytes acted as “Trojan horses,” inducing a positive effect on JE and glia activation and subsequently expanding neuronal infection, causing uncontrolled inflammatory cytokine production and neuronal damage, resulting in the appearance of JE symptoms.

In summary, our results suggest that JEV infection induces the release of HMGB1 from BMECs, enhancing virus-associated leucocyte adhesion and transendothelial migration and promoting viral dissemination, leading to neuroinfection and neuroinflammation (**Figure 7**). Our findings have important implications for the current understanding of JEV-host interactions by highlighting that HMGB1 and monocyte transmigration can be specifically targeted for the treatment of JE.

## Data Availability Statement

The original contributions presented in the study are included in the article/**Supplementary Material**. Further inquiries can be directed to the corresponding author.

## Ethics Statement

The animal study was reviewed and approved by Research Ethics Committee of the College of Veterinary Medicine, Huazhong Agricultural University, Hubei, Wuhan, China.

## Author Contributions

MC and S-SZ designed the investigation. S-SZ, N-YC, Q-CZ, and W-JX performed the experiments. S-SZ, W-JX, KW, and N-YC analyzed the data. MC and S-SZ organized the data. S-SZ and MC wrote the paper. All authors contributed to the article and approved the submitted version.

## Funding

This work was financially supported by the National Program on Key Research Project of China (2016YFD0500406), the Fundamental Research Fund for the Central University (2662018PY016), Natural Science Foundation of Hubei Province (2019CFA010), and the funds of the State Key Laboratory of Agricultural Microbiology.

## Conflict of Interest

The authors declare that the research was conducted in the absence of any commercial or financial relationships that could be construed as a potential conflict of interest.

## Publisher’s Note

All claims expressed in this article are solely those of the authors and do not necessarily represent those of their affiliated organizations, or those of the publisher, the editors and the reviewers. Any product that may be evaluated in this article, or claim that may be made by its manufacturer, is not guaranteed or endorsed by the publisher.

## Abbreviations

BBB, blood-brain barrier; BMEC, brain microvascular endothelial cell; CNS, central nervous system; HBMEC, human brain microvascular endothelial cell; HMGB1, high mobility group box 1; ICAM-1, intercellular adhesion molecule-1; ICAM-2, intercellular adhesion molecule-2; JE, Japanese encephalitis; JEV, Japanese encephalitis virus; LFA-1, lymphocyte function-associated antigen-1; rHMGB1, recombinant HMGB1; TEER, transendothelial electrical resistance; VLA-4, very late antigen-4; VCAM-1, vascular cell adhesion molecule-1.
