# Corrigendum: Brain Microvascular Endothelial Cell-Derived HMGB1 Facilitates Monocyte Adhesion and Transmigration to Promote JEV Neuroinvasion

**DOI:** 10.3389/fcimb.2021.771928

**Published:** 2021-11-11

**Authors:** Song-Song Zou, Qing-Cui Zou, Wen-Jing Xiong, Ning-Yi Cui, Ke Wang, Hao-Xuan Liu, Wen-Juan Lou, Doaa Higazy, Ya-Ge Zhang, Min Cui

**Affiliations:** ^1^ State Key Laboratory of Agricultural Microbiology, College of Veterinary Medicine, Huazhong Agricultural University, Wuhan, China; ^2^ Key Laboratory of Preventive Veterinary Medicine in Hubei Province, The Cooperative Innovation Center for Sustainable Pig Production, Wuhan, China; ^3^ Key Laboratory of Development of Veterinary Diagnostic Products, Ministry of Agriculture of the People’s Republic of China, Wuhan, China; ^4^ International Research Center for Animal Disease, Ministry of Science and Technology of the People’s Republic of China, Wuhan, China

**Keywords:** transmigration, adhesion, monocyte, HMGB1, Japanese encephalitis virus (JEV), neuroinvasion

In the original article, there was a mistake in **Figures 2A, B** as published. **There were spelling errors of the icon of** **Figures 2A, B** ***. “LAF-1” should be “LFA-1”.*** The corrected **Figure 2** appears below.

**Figure 2:**
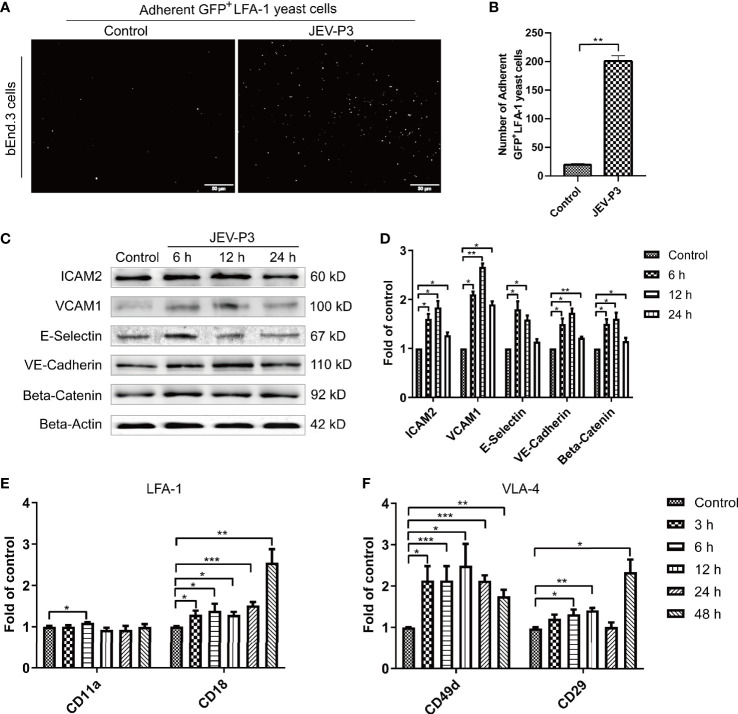
JEV infection upregulated adhesion molecules expression in bEnd.3 cells, and rHMGB1 increased the expression of integrin ligands in splenocytes. **(A)** ICAM-1 expression level was detected in the JEV-infected bEnd.3 cells at 6 h. Representative images showing the binding of highly expressed GFP+ LFA-1 yeast cells. **(B)** Statistical analysis of fluorescence was performed, and the result represents the expression levels of ICAM-1 in bEnd.3 cells. **(C)** Detection of ICAM-2, VCAM-1, E-selectin (CD62E), VE-cadherin, and beta-catenin expression levels in JEV-infected bEnd.3 cells were determined by Western blotting. Protein samples were collected at 0 h (Control), 6 h, 12 h, and 24 h. **(D)** The protein expressions reported in panel **(C)** were normalized to that of beta-actin and quantitatively analyzed as the fold change relative to the control. The expression levels of LFA-1 (CD11a and CD18) **(E)** and VLA-4 (CD49d and CD29) **(F)** in rHMGB1-treated (100 ng/ml) mouse splenocytes, determined by real-time PCR at 0 h (Control), 3 h, 6 h, 12 h, 24 h, 48 h. Untreated cells were served as control. The scale bar for **(A)** is 50 mm. The experiments were repeated at least three times. The data are expressed as the means ± SEM. *p < 0.05, **p < 0.01, and ***p < 0.001.

The authors apologize for this error and state that this does not change the scientific conclusions of the article in any way. The original article has been updated.

In the original article, there was a mistake in **Figures 3A, B** as published. **There were spelling errors of the icon of** **Figures 3A, B**. **“Adhsrent” should be “Adherent”.** The corrected **Figure 3** appears below.

**Figure 3:**
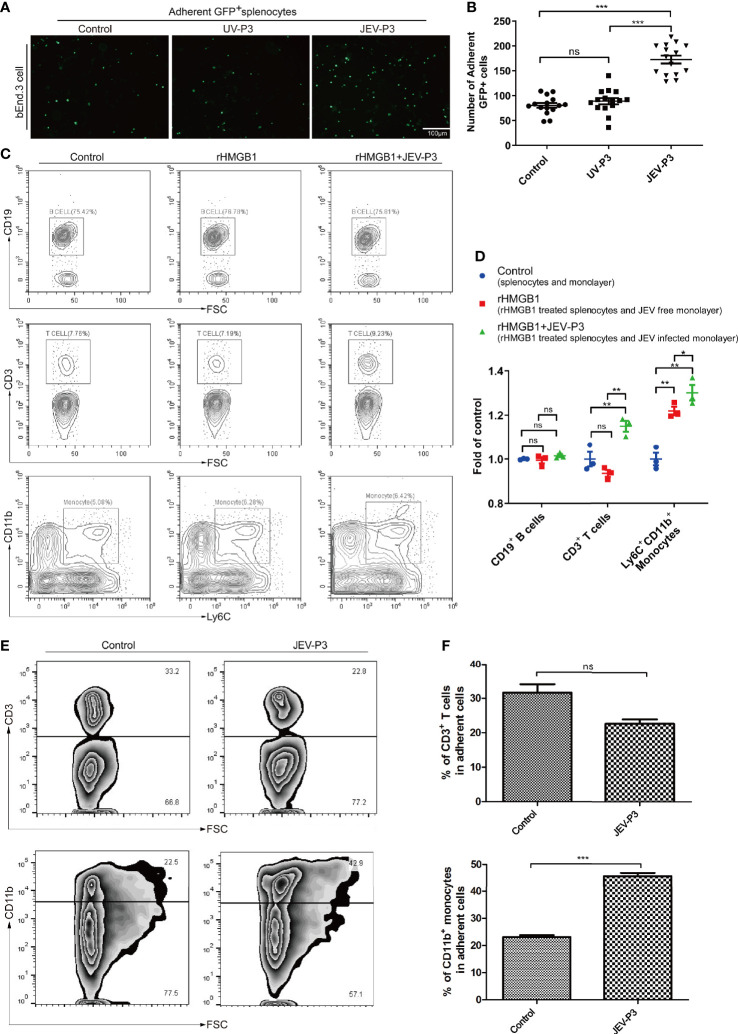
HMGB1 promoted immune cell adhesion to the BMEC monolayer. **(A)** GFP+ splenocytes were incubated with JEV-P3/UV-P3-infected bEnd.3 cell monolayers for 2 h; the GFP+ splenocytes were obtained from transgenic mice. After washing with PBS, the cells were fixed with 4% paraformaldehyde and then observed by fluorescence microscopy. The fluorescence represents the number of adherent splenocytes. JEV free monolayers were served as control. **(B)** Statistical analysis of the GFP+ splenocytes binding to the bEnd.3 cell monolayer of **(A)**. **(C)** Mouse splenocytes were treated with rHMGB1 (100 ng/ml) for 2 h. Then, splenocytes (rHMGB1 treated or untreated) were incubated with the bEnd.3 cell monolayers (JEV infected or uninfected) for 2 h. After gentle washing with PBS, the adherent splenocytes were collected, and the amount of CD19+ B cells, CD3+ T cells, and Ly6C+CD11b+ monocytes was analyzed by flow cytometry. Untreated splenocytes and uninfected monolayers were served as control. **(D)** Statistical analysis of the binding splenocytes to the bEnd.3 cell monolayer as reported in **(C)**. **(E)** Purified CD3+ T cells and CD11b monocytes [obtained from JEV-infected mice, tail vein injection **(F)**, 3 dpi] were inoculated onto the virus-infected bEnd.3 cell monolayer and incubated for 2 h. After washing with PBS, the bound cells were detected by flow cytometry. The right panels show the results of the statistical analysis of CD3+ T cells and CD11b monocytes binding to the bEnd.3 cell monolayer of **(F)**. JEV free bEnd.3 cell monolayers were served as control. The scale bar for **(A)** is 100 mm. These experiments were repeated at least three times. The data are expressed as the means ± SEM. p > 0.05 (ns, no significant difference), *p < 0.05, **p < 0.01, and ***p < 0.001.

The authors apologize for this error and state that this does not change the scientific conclusions of the article in any way. The original article has been updated.

In the original article, there was a mistake in the ***Supplementary material*** as published. **There was an error of the caption of *uncropped*** **Supplementary Figure S1A** **.**“Uncropped **Supplementary Figure 1A**” should be captioned “Uncropped **Figure 1F**”. The corrected **Supplementary material** ***(Uncropped*** **Figure 1F** ***)*** appears below.

**Uncropped Figure 1F:**
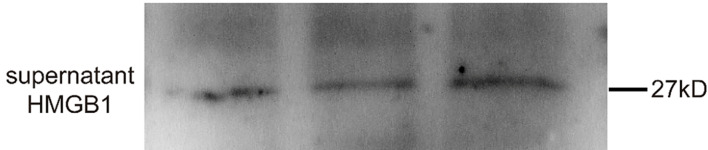


The authors apologize for this error and state that this does not change the scientific conclusions of the article in any way. The original article has been updated.

## Publisher’s Note

All claims expressed in this article are solely those of the authors and do not necessarily represent those of their affiliated organizations, or those of the publisher, the editors and the reviewers. Any product that may be evaluated in this article, or claim that may be made by its manufacturer, is not guaranteed or endorsed by the publisher.

