# Corrigendum: brain microvascular endothelial cell-derived HMGB1 facilitates monocyte adhesion and transmigration to promote JEV neuroinvasion

**DOI:** 10.3389/fcimb.2022.964880

**Published:** 2022-07-29

**Authors:** Song-Song Zou, Qing-Cui Zou, Wen-Jing Xiong, Ning-Yi Cui, Ke Wang, Hao-Xuan Liu, Wen-Juan Lou, Doaa Higazy, Ya-Ge Zhang, Min Cui

**Affiliations:** ^1^ State Key Laboratory of Agricultural Microbiology, College of Veterinary Medicine, Huazhong Agricultural University, Wuhan, China; ^2^ Key Laboratory of Preventive Veterinary Medicine in Hubei Province, The Cooperative Innovation Center for Sustainable Pig Production, Wuhan, China; ^3^ Key Laboratory of Development of Veterinary Diagnostic Products, Ministry of Agriculture of the People’s Republic of China, Wuhan, China; ^4^ International Research Center for Animal Disease, Ministry of Science and Technology of the People’s Republic of China, Wuhan, China

**Keywords:** transmigration, adhesion, monocyte, HMGB_1_, Japanese encephalitis virus (JEV), neuroinvasion

In the original article, there was a mistake in **Figures 5D–E** as published. **Images repeated of** **Figures 5D–E** **.** The corrected **Figure 5** appears below.

**Figure 5:**
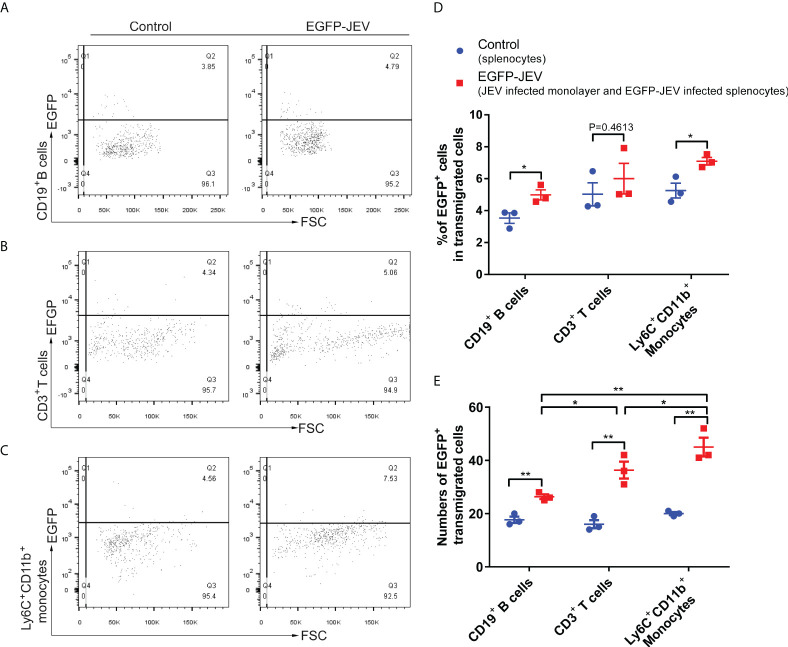
Virus-carrying splenocyte transmigration in vitro. **(A–C)** JEV-infected bEnd.3 cell monolayers were cocultured with EGFP-JEV-infected splenocytes (5 × 105) for 24 h, and the transmigrated cells (lower chamber) were collected and measured by flow cytometry. An enhanced sensitivity measure at 488 nm was performed for the detection of intracellular EGFP-JEV in CD19+ B cells, CD3+ T cells, and Ly6C+ CD11b+ monocytes by flow cytometry. EGFP-JEV free cells were the nonspecific control. **(D, E)** The statistical analysis of EGFP-positive cells in the transmigrated cells in the lower chamber reported in **(A–C)**. The experiments were repeated at least three times. The data are expressed as the means ± SEM. p > 0.05 (ns, no significant difference), *p < 0.05 and **p < 0.01.

In the original article, there was an error in the description *of* **Figure 5D**.

A correction has been made to Results, *Extracellular HMGB1 Facilitated Transendothelial Migration of JEV-Infected Monocytes*, **paragraph 3:

To discover which cells act as virus carriers, JEV with an EGFP tag (EGFP-JEV) was applied to visualize cell transmigration. There was an increased percentage of EGFP-positive Ly6C^+^CD11b^+^ monocytes, CD3^+^ T cells, and CD19^+^ B cells that transmigrated, compared with the control cells (**Figures 5A–C**). Furthermore, there were significantly more transmigrated JEV-positive (EGFP^+^Ly6C^+^CD11b^+^) monocytes than transmigrated JEV-positive T cells (EGFP^+^CD3^+^) or B cells (EGFP^+^CD19^+^) (**Figures 5D, E**).

The authors apologize for these errors and state that this does not change the scientific conclusions of the article in any way. The original article has been updated.

## Conflict of interest

The authors declare that the research was conducted in the absence of any commercial or financial relationships that could be construed as a potential conflict of interest.

## Publisher’s Note

All claims expressed in this article are solely those of the authors and do not necessarily represent those of their affiliated organizations, or those of the publisher, the editors and the reviewers. Any product that may be evaluated in this article, or claim that may be made by its manufacturer, is not guaranteed or endorsed by the publisher.

