# Supplementary material for: Brain Microvascular Endothelial Cell-Derived HMGB1 Facilitates Monocyte Adhesion and Transmigration to Promote JEV Neuroinvasion
Source: Front Cell Infect Microbiol. 2021 Aug 31;11:701820. doi: 10.3389/fcimb.2021.701820 (PMC8439198; doi:10.3389/fcimb.2021.701820)

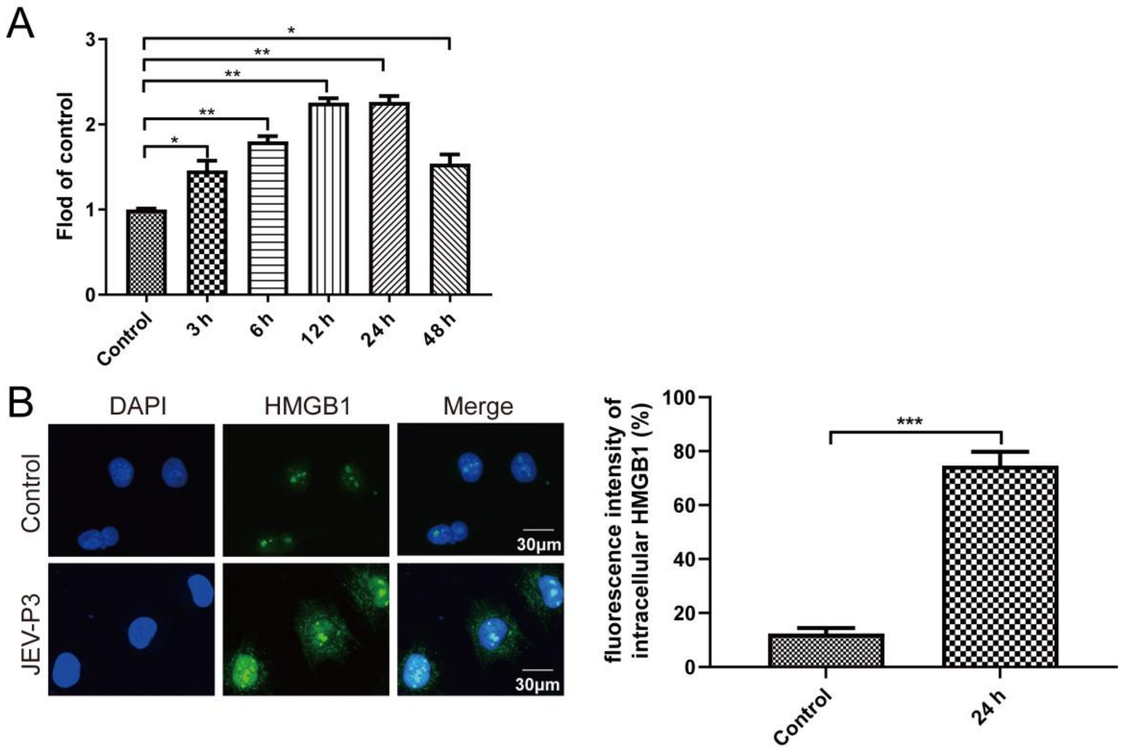

Supplementary Figure 1 Detection of HMGB1 expression in bEnd.3 cells and translocation in HBMECs. HBMECs and bEnd.3 cells were exposed to JEV at an MOI of 1. RNA samples were collected at the indicated times (0 h, 3 h, 6 h, 12 h, 24 h, and 48 h). (**A**) Expression level of HMGB1 in JEV-infected bEnd.3 cells, determined by real-time PCR at 0 h (Control), 3 h, 6 h, 12 h, 24 h, and 48 h. (**B**) HBMECs were infected with JEV-P3. After 24 h, the cells were incubated with anti-HMGB1 polyclonal antibody followed by FITC-conjugated secondary antibody, and then imaged by the confocal microscopy (Nikon, Japan) for DNA (blue, DAPI), and HMGB1 (green). The right panel shows the statistical analysis of fluorescence intensity of intracellular HMGB1. Cells not subjected to viral infection were the control. The scale bar for (A) is 30 μm. These experiments were repeated at least three times. The data are expressed as the means ± SEM. *p < 0.05; **p < 0.01, and ***p < 0.001.

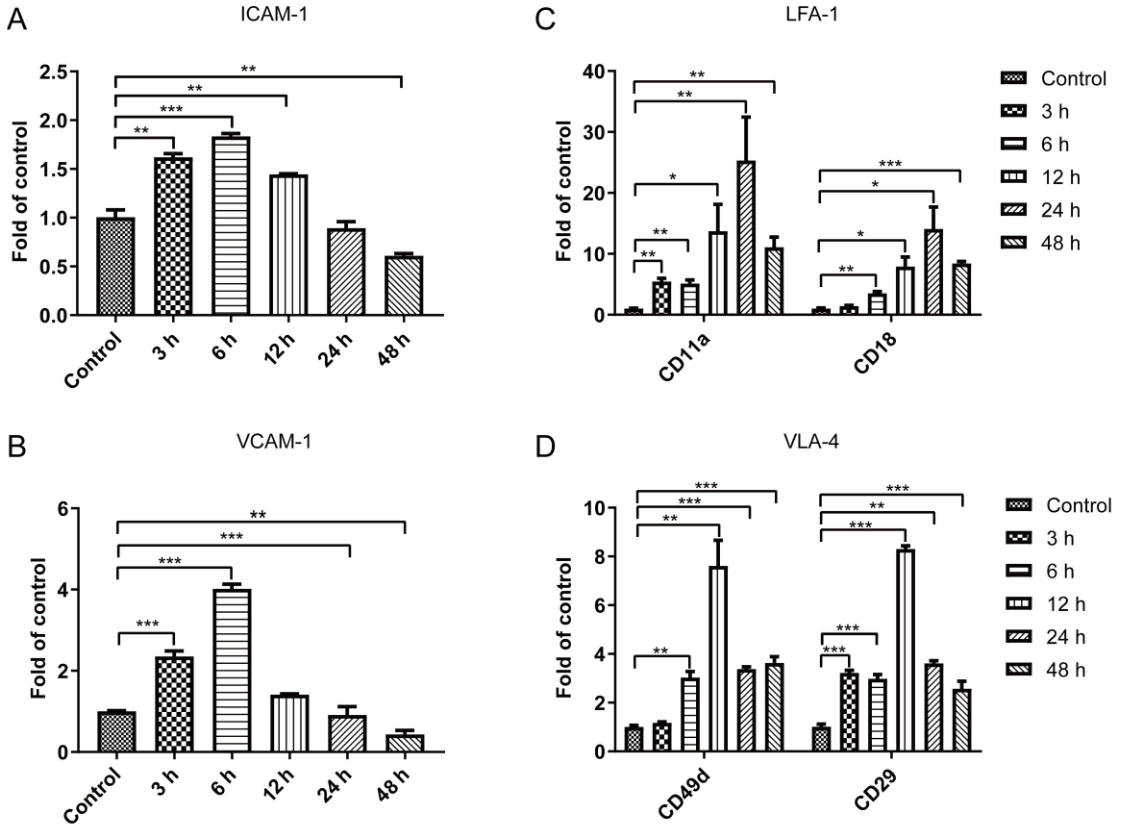

Supplementary Figure 2 JEV regulated adhesion molecules in HBMECs and rHMGB1 increased the expression of integrin ligands in THP-1 cells. HBMECs were exposed with JEV at an MOI of 1. Then, RNA samples were collected at 0 h (Control), 6 h, 12 h, 24 h, and 48 h. Expression of ICAM-1 (**A**) and VCAM-1 (**B**) in HBMECs exposed to JEV-P3 at an MOI of 1 was measured by real-time PCR, with beta-actin as the control. The expression of LFA-1 (CD11a and CD18) (**C**) and VLA-4 (CD49a and CD29) (**D**) were measured in recombinant HMGB1 (100 ng/ml) treated THP-1 cells at 0 h (Control), 6 h, 12 h, 24 h, and 48 h. The experiments were repeated at least three times. The data are expressed as the means ± SEM. *p < 0.05; **p < 0.01, and ***p < 0.001.

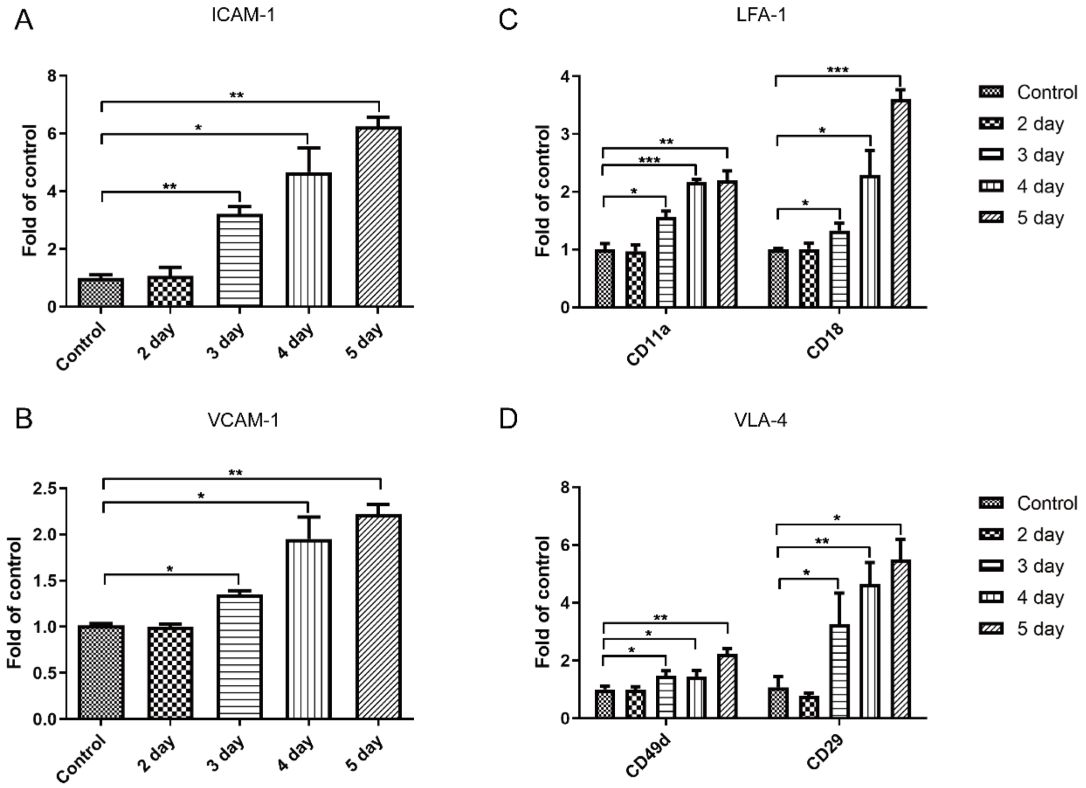

Supplementary Figure 3 Effects of JEV on adhesion molecules and integrin ligands in vivo. Mice were infected with 5.0×10^6 PFU of JEV via intravenous injection (i.v.). Brains and PBMCs were collected at the indicated times (day 0, day 2, day 3, day 4, and day 5). Expression levels of ICAM-1 (**A**) and VCAM-1 (**B**) in JEV-infected brains, and their ligands LFA-1 (CD11a and CD18) (**C**) and VLA-4 (CD49d and CD29) (**D**) in JEV-infected PBMCs, which were detected by real-time PCR at indicated times. Uninfected mice were served as control.The experiments were repeated at least three times. The data are expressed as the means ± SEM. *$p < 0.05$; **$p < 0.01$, and ***$p < 0.001$.

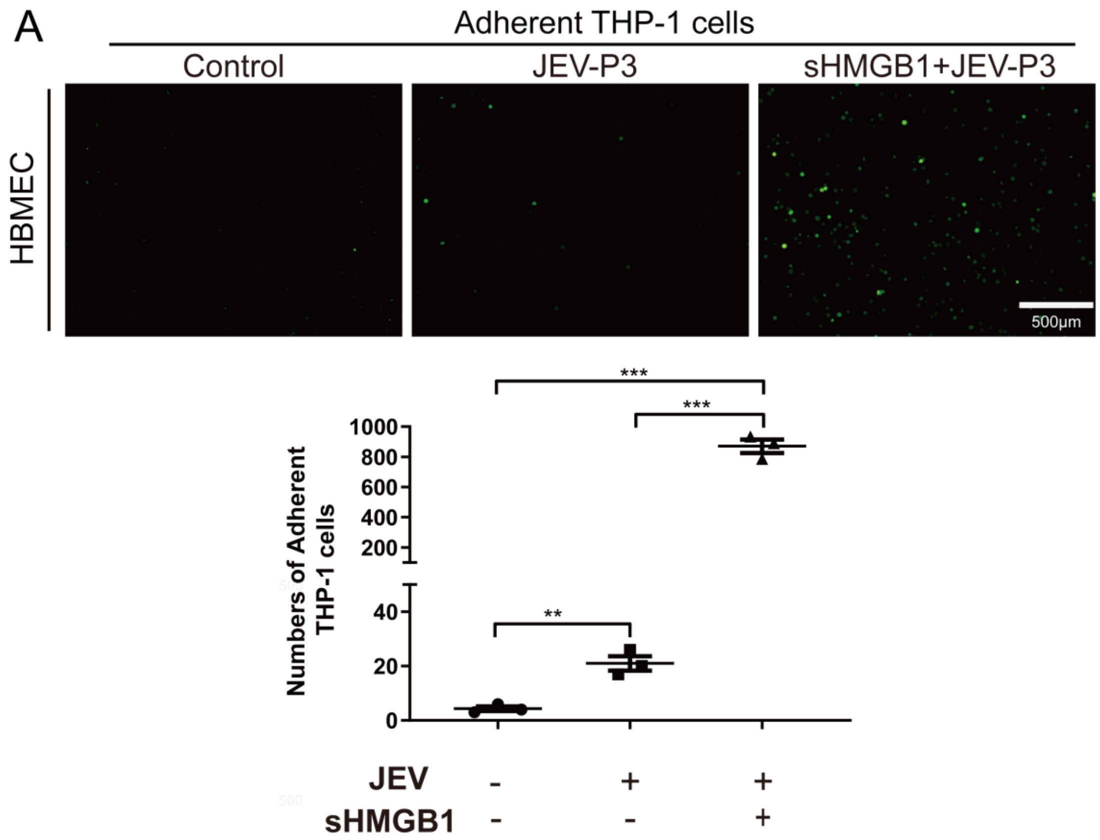

Supplementary Figure 4 HMGB1 promoted immune cell adhesion to the BMEC monolayer. (**A**) HBMECs were exposed with JEV at an MOI of 1. THP-1 cells were treated with sHMGB1 (supernatant HMGB1). HBMEC were incubated with THP-1 cells and mice splenocytes for 2 h. The monolayers were washed with PBS, fixed with 4% paraformaldehyde, immunostained with anti-CD14 (Abcam, USA) and FITC-labeled secondary antibodies (Invitrogen, USA), and then observed by fluorescence microscope. JEV free and sHMGB1 free were served as control. The scale bar for (**A**) is 500 μm. The experiments were repeated at least three times. The data are expressed as the means ± SEM. $^{**}p < 0.01$, and $^{***}p < 0.001$.

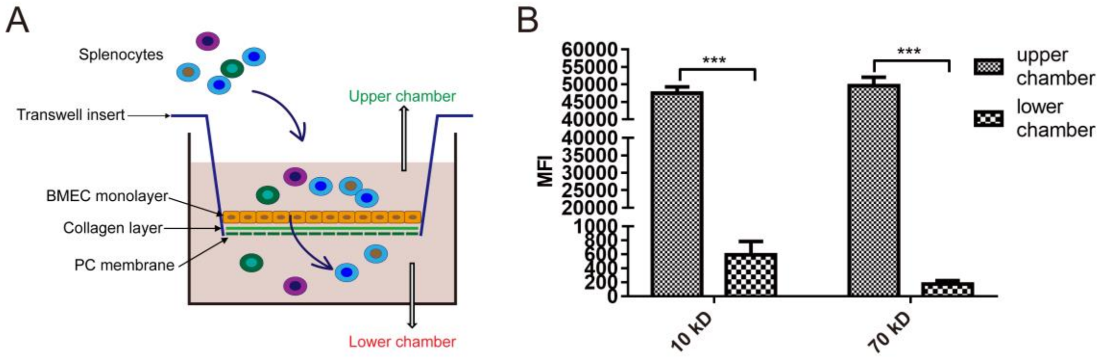

Supplementary Figure 5 Permeability evaluation of the Transwell model. (A) Scheme of the Transwell migration assay. (B) FITC-dextran (10 kD and 70 kD) was added in the upper chamber. After 30 min, the mediums were collected (from the upper chamber and lower chamber) for the detection of the fluorescence intensity by ELIASA. The experiments were repeated at least three times. The data are expressed as the means ± SEM. ***$p < 0.001$.

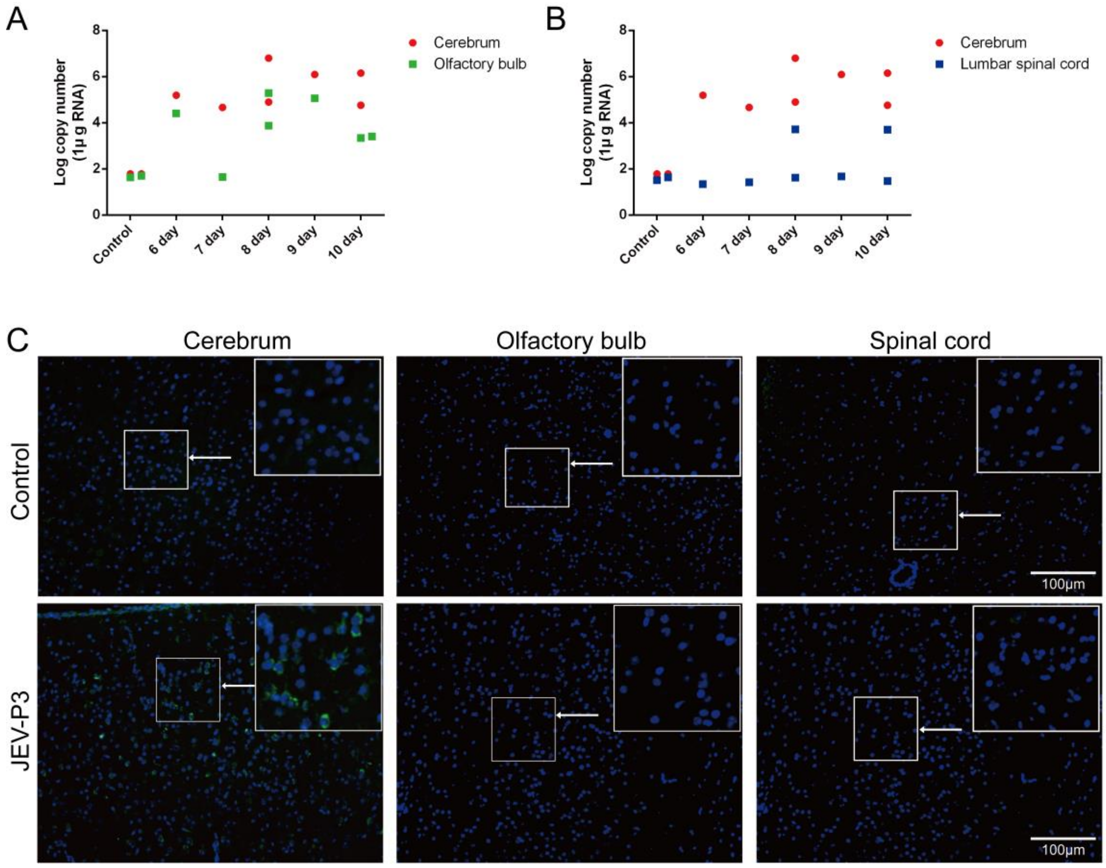

Supplementary Figure 6 Distribution of JEV in the brain, olfactory bulb and spinal cord of JE mice. Cerebra, olfactory bulbs and spinal cords were collected from JEV-infected mice (footpad injection) at the indicated times (day 6, day 7, day 8, day 9, and day 10). (**A**) (**B**) JEV was detected by RT-PCR at the indicated times in the cerebrum, olfactory bulb and spinal cord. (**C**) JEV accumulation was detected in the cerebrum, olfactory bulb and spinal cord on day 7 postinfection and imaged by immunofluorescence staining for DNA (blue, DAPI) and JEV (green, JEV-E protein). Uninfected mice were served as control. The scale bar for (**C**) is 100 μm.

Table 1 Sequences of real-time PCR primers used in this study.

**mRNA**	**Primers**	**Sequence (5′-3′)**
Human-β-actin	Upstream	CGTGCGTGACATTAAGGAGAA
	Downstream	GGAAGGAAGGCTGGAAGAGT
Mouse-β-actin	Upstream	CCTGTATGCCTCTGGTCGTA
	Downstream	CTGTGGTGGTGAAGCTGTAG
Human-HMGB1	Upstream	TATGGCAAAAGCGGACAAGG
	Downstream	CTTCGCAACATCACCAATGGA
Mouse-HMGB1	Upstream	AGATATGGCAAAGGCTGACAAGGC
	Downstream	GGGCGGTACTCAGAACAGAACAAG
JEV-C	Upstream	GGCTCTTATCACGTTCTTCAAGTTT
	Downstream	TGCTTTCCATCGGCCYAAAA
Mouse-ICAM	Upstream	CGTGCTGTATGGTCCTCG
	Downstream	GGGCTTGTCCCTTGAGTT
Mouse-VCAM	Upstream	TTGGGGGCAACGTTGACATA
	Downstream	TGAAGGGGGAGACTACACTGA
Mouse-CD11a	Upstream	CTCTCCGTCTCCTTCAACAG
	Downstream	GCCTCCATCTTCTCCTTCAG
Mouse-CD18	Upstream	AAAGTGACACTTTACTTGCGAC
	Downstream	GAGGTAGTACAGATCAATGGGG
Mouse-CD49a	Upstream	GGAAGAGAGTGTACATTTGCAC
	Downstream	ACAGTGTTGATCAGCTTTCATG
Mouse-CD29	Upstream	TACTCTGGAAAATTCTGCGAGT
	Downstream	ATAGCATTCACAAACACGACAC
Human-ICAM1	Upstream	TGCAAGAAGATAGCCAACCAAT
	Downstream	GTACACGGTGAGGAAGGTTTTA
Human-VCAM1	Upstream	CAGGCTGGAGATAGACTTACTG
	Downstream	CCTCAATGACAGGAGTAAAGGT
Human-CD11a	Upstream	CAGATTGGCTCTTATTTCGGTG
	Downstream	TTCTCTGGTAGATAAACACCCG
Human-CD18	Upstream	CTCTCCTACTCCATGCTTGAT
	Downstream	CACGGTCTTGTCCACGAA
Human-CD49a	Upstream	TTCGGAGCCAGCATACTACC
	Downstream	ATTGAGGTCCACAGCACAGA
Human-CD29	Upstream	CTGTGATGCCTTACATTAGCAC
	Downstream	ATCCAAATTTCCAGATATGCGC

Uncropped Figure 1B

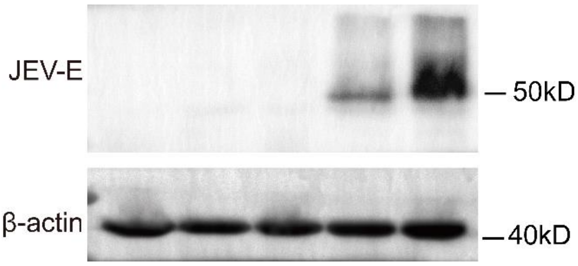

Uncropped Figure 1D

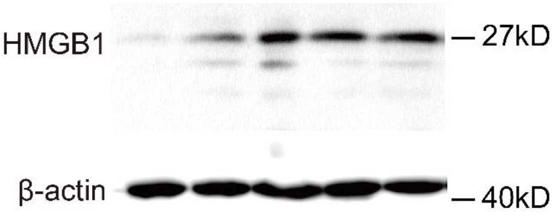

Uncropped Figure 1E

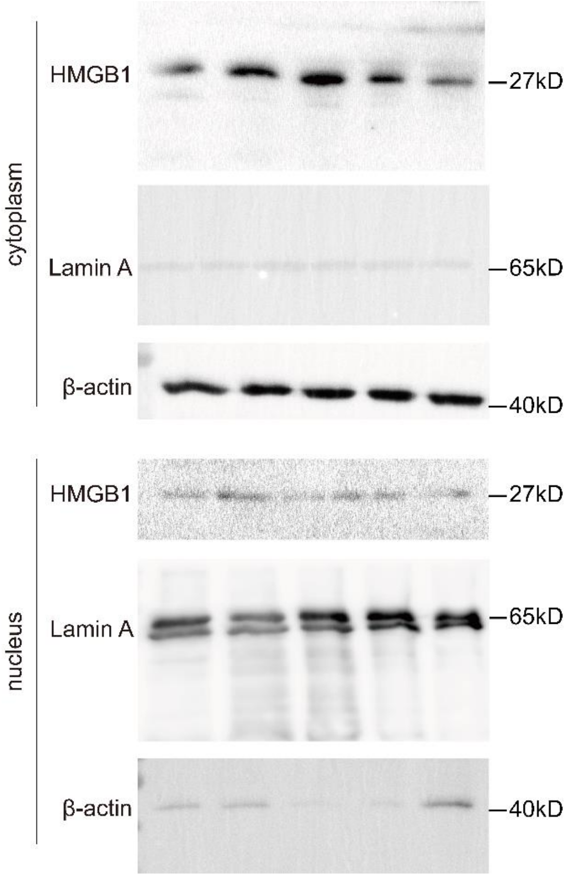

Uncropped Figure 1F

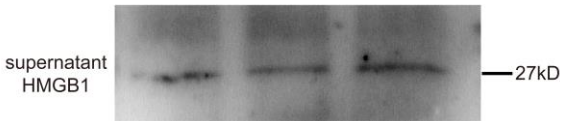

Uncropped Figure 2C

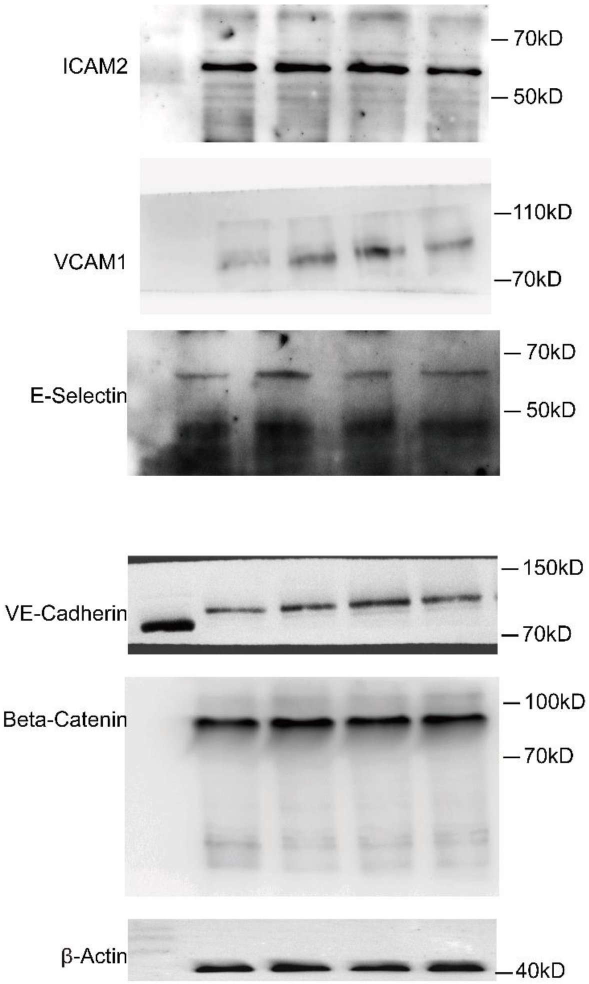

Uncropped Figure 6B

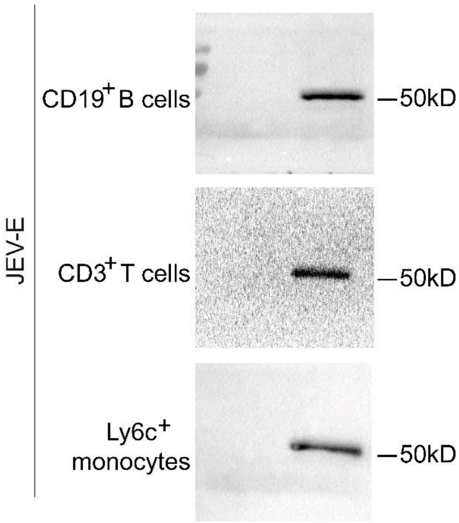

Supplement: Supplementary file 1 [file DataSheet_1.pdf]
